# New Approaches on Quantification of *Campylobacter jejuni* in Poultry Samples: The Use of Digital PCR and Real-time PCR against the ISO Standard Plate Count Method

**DOI:** 10.3389/fmicb.2017.00331

**Published:** 2017-03-02

**Authors:** Bojan Papić, Mateja Pate, Urška Henigman, Urška Zajc, Igor Gruntar, Majda Biasizzo, Matjaž Ocepek, Darja Kušar

**Affiliations:** ^1^Veterinary Faculty, Institute of Microbiology and Parasitology, University of LjubljanaLjubljana, Slovenia; ^2^Veterinary Faculty, Institute of Food Safety, Feed and Environment, University of LjubljanaLjubljana, Slovenia

**Keywords:** *Campylobacter jejuni*, poultry, quantification, plate counting, qPCR, dPCR

## Abstract

Campylobacteriosis is the most frequently reported bacterial food-borne illness in the European Union and contaminated broiler meat is considered the most important source of infection in humans. The aim of the present study was to evaluate real-time PCR (qPCR) and digital PCR (dPCR) for quantification of *Campylobacter jejuni* in 75 broiler neck-skin samples collected from a poultry slaughterhouse, and to compare them with the ISO 10272-2 standard plate count method. For qPCR standard curve, *C. jejuni*-negative neck-skin samples were spiked with *C. jejuni* suspension with a known number of bacterial cells. The observed CFU/g values by qPCR correlated greatly with the expected values and qPCR showed good performance with the reliable limit of detection (rLOD) and limit of quantification (LOQ) of three and 31 target copies per reaction, respectively. However, both rLOD (1219 CFU/g) and LOQ (12,523 CFU/g) were beyond the EFSA-proposed critical limit of 500–1,000 CFU/g of neck skin. Although *C. jejuni* cell counts were ≤1,000 CFU/g in only 7/75 samples by plate counting, they were ≤LOQ in 60/75 and ≤rLOD in 26/75 (≤1,000 CFU/g in 24/75) samples by qPCR. A strong and statistically significant correlation was observed between qPCR and dPCR. Both PCR-based methods correlated significantly with the plate count method; however, the correlation was moderate. Using the Bland–Altman analysis, an average agreement was noted between all three methods, although with a large standard deviation. A significant bias toward overestimation in dPCR was observed, probably due to the relatively high number of false positive calls. The linear dynamic range was comparable in both PCR-based methods; however, qPCR proved to be more suitable for routine use. In the future, the establishment of a reliable molecular quantification of *C. jejuni* in poultry samples showing a wide range of contamination levels down to the proposed critical limit is needed to enable time- and cost-effective surveillance throughout all stages in the food production chain. As both rLOD and LOQ were beyond this limit, a modification of the procedure is suggested to include less sample dilution prior to DNA extraction to enable PCR-based quantification of *C. jejuni* at the proposed microbiological criteria.

## Introduction

*Campylobacter* spp. has been the most commonly reported bacterial gastrointestinal pathogen in humans in the European Union (EU) since 2005. The EU notification rate increased by 10% in 2014, compared with the previous year, and a statistically significant increasing trend was observed in the 7-year period from 2008 to 2014 (EFSA, 2015). The main causative agent of intestinal campylobacteriosis is *Campylobacter jejuni*, which is naturally present in the intestines of birds, especially in industrial poultry production. Contaminated broiler meat is considered the most important source of infection in humans (EFSA, 2015). Contamination of poultry meat occurs during the slaughterhouse processing (Berrang et al., 2001); as campylobacters persist throughout the food chain, they represent a public health risk. According to EFSA estimates, the latter could be considerably reduced if all slaughtered poultry batches would comply with microbiological criteria with a critical limit of 1,000 or 500 CFU/g of neck and breast skin (Debretsion et al., 2007). To reliably quantify the extent of *C. jejuni* contamination in such samples, appropriate (rapid, accurate, reliable, and reasonably priced) enumeration methods should be used.

Rapid and reliable detection and quantification of the organism directly in samples remain challenging. Traditional plate counting method is a time consuming procedure, requiring several working days to be completed (ISO 10272-2:2006; ISO, 2006a). Its advantage, but possibly also one of its biggest drawbacks, is that it only recovers cells which can be readily cultivated *in vitro*. Along with the dead or damaged bacteria, the presence of viable but non-culturable (VBNC) cells, which can occur as an adaptation to environmental stress, may lead to underestimation of *Campylobacter* numbers in investigated samples (Thomas et al., 2002). Nevertheless, the ISO 10272-2:2006 plate counting method is currently the only acceptably validated and standardized *Campylobacter* quantification method, thus allowing the comparison of results between different laboratories and matrices.

Molecular methods such as real-time PCR (also called quantitative PCR; qPCR) provide certain advantages in *C. jejuni* quantification, especially in terms of turnaround time, specificity and sensitivity, and have already been used for different applications related to poultry industry, e.g., for quantification in poultry carcass rinses (Debretsion et al., 2007; Botteldoorn et al., 2008), fecal and cecal samples (Rudi et al., 2004; Garcia et al., 2013), carcasses (Melero et al., 2011; Ivanova et al., 2014), neck-skin samples (Schnider et al., 2010), and samples from the slaughtering environment (Melero et al., 2011; Ivanova et al., 2014). The third generation of PCR technology—digital PCR (dPCR)—is reported to offer further advantages in precise quantification of nucleic acids in terms of higher sensitivity and reproducibility compared to qPCR (Strain et al., 2013; Taylor et al., 2015). One of major advantages of dPCR is the absolute quantification with no calibration curve required (Huggett et al., 2015). Chip-based dPCR is supported by the endpoint PCR assay in which a sample is diluted and partitioned into thousands of separate reaction chambers to each contain one or no copies of the target sequence. Calculation of the absolute quantities is based upon counting the number of positive vs. negative partitions at an appropriate dilution level to comply with Poisson statistics that sustains the algorithms behind dPCR quantification (Baker, 2012). Nevertheless, one of the major drawbacks of DNA-based techniques used for quantification is their inability to distinguish between DNA from viable and dead cells (Nocker and Camper, 2006), which is probably the most important obstacle in implementation of these methods in routine applications.

The aim of the present study was to evaluate two molecular methods, qPCR and dPCR, for *C. jejuni* quantification in the naturally contaminated broiler neck-skin samples collected at the slaughterhouse, and to compare them with the ISO 10272-2:2006 standard-based plate counting method. To the best of our knowledge, this is the first report on the use of dPCR for *C. jejuni* quantification in poultry samples.

## Materials and methods

### Samples

#### Standard curve samples

For validation of qPCR and dPCR, *C. jejuni* ATCC 33,560 was cultivated in the brain heart infusion (BHI) broth (Oxoid, UK) at 37°C to obtain an overnight culture containing 1.1 × 10^8^ CFU/g as determined by the plate count method. A 10-fold dilution series was prepared in BHI. Poultry neck-skin samples (1 g) that tested negative for the presence of *C. jejuni* by the plate count method and qPCR were spiked with 100 μl of dilution series. Dilutions ranging from 1.1 × 10^7^ CFU/ml (dilution 10^0^) to 1.1 CFU/ml (dilution 10^−7^) were obtained and samples processed as later employed for the naturally contaminated poultry neck skin. All dilutions were spiked in triplicates (biological replicates) and subjected to DNA extraction prior to qPCR (in three technical replicates) and dPCR (in one technical replicate).

#### Broiler neck-skin samples

Sixty individual and 15 pooled (a pool consisted of neck-skin samples from 10 broilers) neck-skin samples were collected in the scope of two research projects aiming to reduce *Campylobacter* contamination levels at slaughterhouses. Approximately 1 g of neck skin from each individual carcass was taken to analyze the contamination level of *C. jejuni* according to ISO 10272-2:2006 method (ISO, 2006a). Briefly, pooled (10 × 1 g) and non-pooled skin samples (10 g each) were supplemented with 90 ml of buffered peptone water and homogenized (10-fold dilutions). One milliliter of suspension was used for DNA extraction and 1 ml for enumeration by the plate count method.

### Enumeration

#### Enumeration i: plate count method

Enumeration and determination of *C. jejuni* in 1 ml of initial suspension (skin homogenate or culture) were performed according to ISO 10272:2006 methods (ISO, 2006a,b). For enumeration of *C. jejuni* culture, 1 ml of initial culture suspension was applied onto three mCCDA (modified charcoal cefoperazone desoxycholate agar; Oxoid, UK) plates and 0.1 ml of further decimal dilutions up to 10^−8^ on single mCCDA plates. For enumeration of skin-homogenate samples, 1 ml of sample and decimal dilutions to 10^−3^ were spread plated. After 40–48 h incubation at 41.5°C in the microaerophilic atmosphere created by the GENbox generators (BioMerieux, France), plates from two successive dilutions with <150 *Campylobacter*-suspected colonies per plate were counted to obtain the final colony forming units (CFU) per unit of measure (g or ml). To determine the isolates to the species level, the hippurate and indoxyl acetate hydrolysis, catalase and susceptibility to cephalotine and nalidixic acid tests were performed. Two suspected *Campylobacter* colonies from each sample were randomly selected for identification.

#### DNA extraction

Total DNA was extracted from 1 ml of the prepared samples (24 standard curve samples, 60 individual, and 15 pooled skin homogenates) using Isolation from Complex Samples Kit (Institute of Metagenomics and Microbial Technologies, Slovenia) according to the manufacturer's instructions with minimal adjustments described by Logar et al. (2012). All DNA samples were stored at −20°C until use.

#### Enumeration II: qPCR

For quantification of *C. jejuni* by qPCR, a standard curve analysis was performed to validate the procedure. The negative matrix was spiked in three biological replicates with an overnight *C. jejuni* culture diluted in 10-fold series as described above. After DNA extraction, all biological replicates were tested with qPCR in three technical replicates to calibrate the qPCR assay adopted from Toplak et al. (2012) prior to the in-house use. Amplification efficiency of the reaction was calculated according to the equation *E* = 10^−1/slope^ − 1. For the calculation of standard curve equation, only data belonging to the linear dynamic range was considered (coefficient of variation <33%; Žel et al., 2012). Results were reported in C_q_ values. Limit of quantification (LOQ) and C_q_ cut-off value were determined as described by Kušar et al. (2013). Reliable limit of detection (rLOD) was determined where at least 95% of positive replicates were detected, and limit of detection (LOD) as the lowest concentration of *C. jejuni* with at least one positive replicate. In brief, the assay variability expressed by the coefficient of variation (CV) for each standard dilution was determined as the standard deviation of the calculated concentrations (from C_q_ values of nine replicates according to the equation of fitted regression line) in proportion to the average calculated concentration. According to the obtained CV values, LOQ was determined as CVs are markedly larger below the PCR quantification limit (Vaerman et al., 2004). Reliable LOD was set accordingly lower, i.e., 5- to 10-fold lower than LOQ in complex samples (Berdal and Holst-Jensen, 2001). For determination of the C_q_ cut-off value, the highest C_q_ was considered belonging to the first standard dilution of the dilution series where no amplification was observed in some of the replicates; this C_q_ was rounded up to the next half value and 0.5 was added to obtain the C_q_ cut-off value (Mehle et al., 2012).

The 25-μl qPCR reactions contained 2.5 μl DNA, 1 × TaqMan Universal PCR Master Mix (Applied Biosystems by Thermo Fisher Scientific, USA) and *ccoN* primers and probe concentrations at previously described concentrations (Toplak et al., 2012). Besides the previously tested specificity on two phylogenetically related and ten unrelated species (Toplak et al., 2012), the specificity of the assay was additionally tested on *Campylobacter* species that serve as controls for routine PCR assays (*Campylobacter coli, C. lari, C. upsaliensis, C. fetus* subsp. *fetus, C. fetus* subsp. *venerealis*). Thermal amplification was performed in AB 7500 Fast Real-Time PCR System (Applied Biosystems by Thermo Fisher Scientific, USA).

#### Enumeration III: dPCR

Prior to implementation, dPCR was validated using the same standard curve samples as for qPCR validation. Quality threshold (Q_T_) and fluorescence threshold (F_T_) were determined based on the comparison of 14 negative template controls (i.e., negative matrix controls as determined by the plate count method and qPCR; NTC), 10 water no template controls (PCR grade water, WNTC), and 10 positive controls for increased stringency. The observed copy number/μl in dPCR was converted to CFU/ml taking into account all dilution factors.

Absolute quantification of *C. jejuni* with dPCR was performed in 15-μl reactions consisting of 7.5 μl QuantStudio 3D Digital PCR Master Mix v2 (Applied Biosystems by Thermo Fisher Scientific, USA), 3 μl DNA, 3.75 μl of PCR grade water, and *ccoN* primers and probes at previously described concentrations (Toplak et al., 2012). A 14.5 μl aliquot of each reaction mix was loaded onto dPCR chips from QuantStudio 3D Digital PCR 20K Chip Kit v2 using QuantStudio 3D Digital PCR Chip Loader. Amplification was performed in the PCR cycler ProFlex 2 × flat PCR System (Applied Biosystems by Thermo Fisher Scientific, USA) according to manufacturer's instructions. NTC and a positive control were included in each run. After amplification, all chips were analyzed using QuantStudio 3D AnalysisSuite 3.0.3.

#### Statistics

All numbers were log_10_ transformed to ensure the data was normally distributed. Regression analysis and Bland–Altman plots were performed using MedCalc v17.1 (MedCalc Software, Belgium). *P* ≤ 0.05 was considered statistically significant.

## Results

### Validation of qPCR

**Figure 1:**
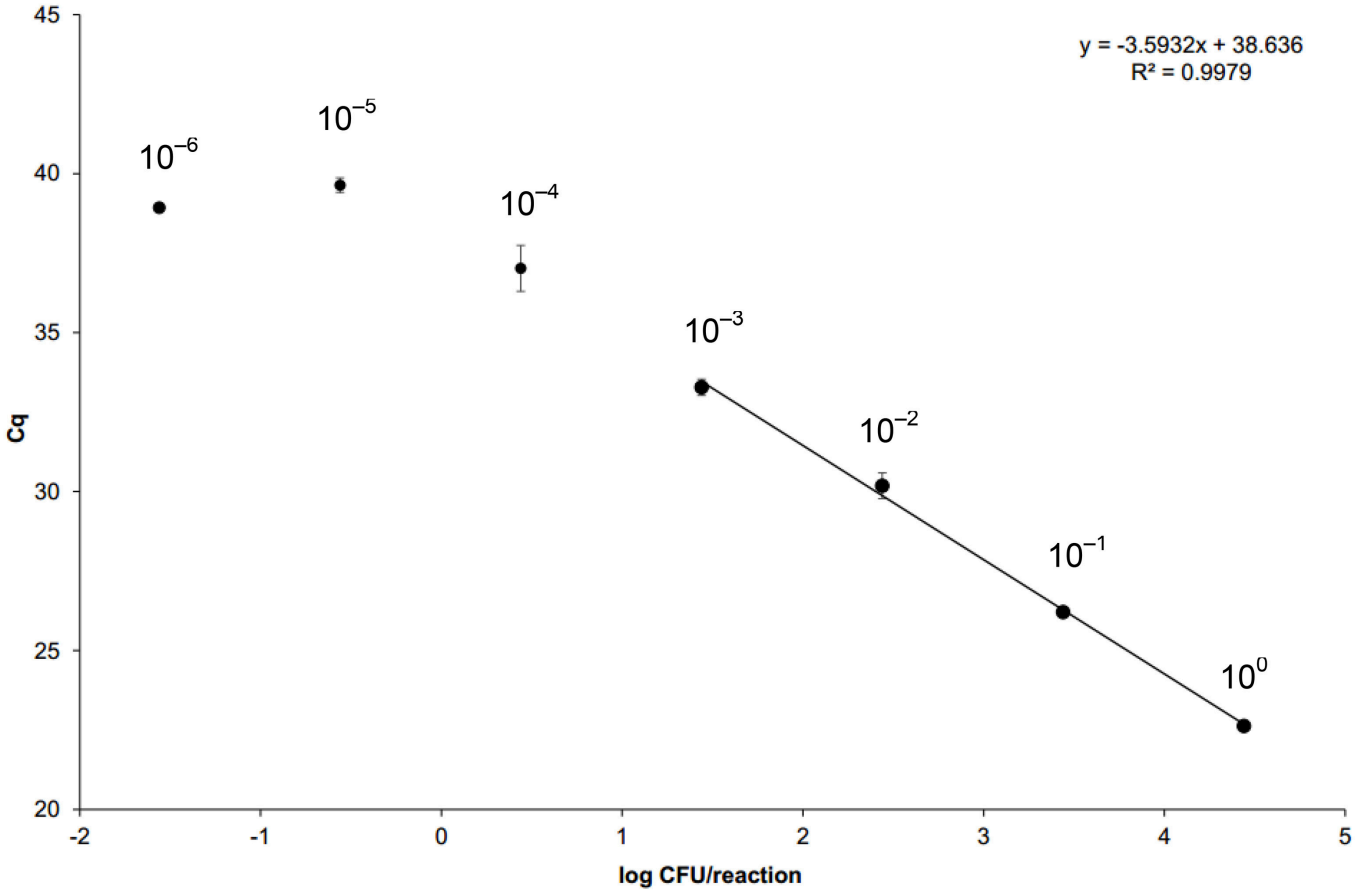
**Standard curve for qPCR based on average cycle of quantification (C_q_) values plotted against the estimated number of target copy number per reaction**. Negative poultry neck-skin samples were spiked with 100 μl of 10-fold dilution series, prepared from the overnight *Campylobacter jejuni* culture (1.1 × 10^8^ CFU/ml) and ranging from 1.1 × 10^7^ CFU/ml (dilution 10^0^) to 1.1 CFU/ml (dilution 10^−7^). All dilutions were spiked in triplicates (biological replicates) and samples processed as employed for the naturally contaminated poultry neck skin. After DNA extraction, all biological replicates were tested with qPCR in three technical replicates. Amplification efficiency (*E* = 10^−1/slope^ − 1) of the reaction was 89.80%. For the calculation of standard curve equation, only data belonging to the linear dynamic range was considered (coefficient of variation <33%). Dilutions 10^−7^ gave negative results. Error bars represent standard deviations of averaged C_q_s. For raw data, see Table 1.

The in-house standard curve of 10-fold dilutions of standard DNA extracted from *C. jejuni*-spiked negative poultry neck skin is shown in Figure 1 with regression-curve equation and regression coefficient *R*^2^. The amplification efficiency was 89.80%. The C_q_ cut-off value was set to 40.5 according to the first standard dilution with no amplification in at least one replicate (dilution 10^−5^ in Table 1). In this study, 2/3 replicates were negative for the particular limiting dilution 10^−5^ (CV = 151.46%) and LOD was calculated from the obtained C_q_ values (<3 CFU/reaction, 70 CFU/g).

**Table 1:** **In-house calibration of qPCR for *Campylobacter jejuni* (qPCR assay adopted from Toplak et al., 2012)**.

**Biological replicate**	**Dilution**	**Expected CFU/reaction**	Average Cq^*^	**Average observed CFU/reaction ±*SD***	**CV (%)**
1	10^−0^	27,500	22.69 (3/3)	27,506 ± 1,908	6.94
2	10^−0^	27,500	22.56 (3/3)	29,902 ± 1,476	4.94
3	10^−0^	27,500	22.64 (3/3)	28,384 ± 2,113	7.44
Average	10^−0^	27,500	22.63 (9/9)	28,597 ± 1,916	6.70
1	10^−1^	2,750	26.24 (3/3)	2,648 ± 79	2.99
2	10^−1^	2,750	26.11 (3/3)	3,072 ± 155	5.05
3	10^−1^	2,750	26.19 (3/3)	2,915 ± 139	4.77
Average	10^−1^	2,750	26.21 (9/9)	2,878 ± 217	7.53
1	10^−2^	275	30.33 (3/3)	210 ± 53	25.32
2	10^−2^	275	30.15 (3/3)	236 ± 58	24.66
3	10^−2^	275	30.07 (3/3)	250 ± 72	29.03
Average	10^−2^	275	30.18 (9/9)	232 ± 56	24.29
1	10^−3^	27.5	33.50 (3/3)	27 ± 6	20.96
2	10^−3^	27.5	33.12 (3/3)	34 ± 4	10.82
3	10^−3^	27.5	33.22 (3/3)	32 ± 3	9.55
Average	10^−3^	27.5	33.28 (9/9)	31 ± 5	15.59
1	10^−4^	2.75	37.10 (3/3)	3 ± 1	27.04
2	10^−4^	2.75	37.41 (3/3)	3 ± 1	54.96
3	10^−4^	2.75	36.55 (3/3)	4 ± 1	23.81
Average	10^−4^	2.75	37.02 (9/9)	3 ± 1	36.36
1	10^−5^	0.275	39.47 (1/3)	<3	
2	10^−5^	0.275	39.90 (1/3)	<3	
3	10^−5^	0.275	39.54 (1/3)	<3	
Average	10^−5^	0.275	39.64 (3/9)	<3	151.46
1	10^−6^	0.0275	/ (0/3)		
2	10^−6^	0.0275	/ (0/3)		
3	10^−6^	0.0275	38.93 (1/3)	<3	
Average	10^−6^	0.0275	38.93 (1/9)	<3	
1	10^−7^	0.00275	/ (0/3)		
2	10^−7^	0.00275	/ (0/3)		
3	10^−7^	0.00275	/ (0/3)		

* *number of positive/all technical replicates. Expected CFU/reaction is based on the results of the plate count method, assuming no loss during processing. SD, standard deviation; CV, coefficient of variation*.

According to CVs, LOQ was set to ~31 CFU/reaction (dilution 10^−3^ in Table 1). Assuming no losses in DNA extraction and taking into account the volume of the elution buffer, this corresponded to 1,252 CFU/ml of poultry skin homogenate. According to the obtained C_q_ values for dilution series and calculated concentrations, rLOD was set to ~3 CFU/reaction (dilution 10^−4^ in Table 1), which is the most sensitive reliable LOD theoretically possible (Bustin et al., 2009) and was in congruence with the theoretical 5- to 10-fold difference between LOQ and rLOD (Berdal and Holst-Jensen, 2001). The obtained rLOD translated to 122 CFU/ml. Due to the sample preparation procedure that included supplementation of skin samples with 10-times the volume of buffered peptone water and homogenization, the obtained LOQ and rLOD in CFU/g should be multiplied by 10 to obtain contamination levels for chicken skin samples: 12,523 CFU/g (LOQ) and 1,219 CFU/g (rLOD).

### Validation of dPCR

**Figure 2:**
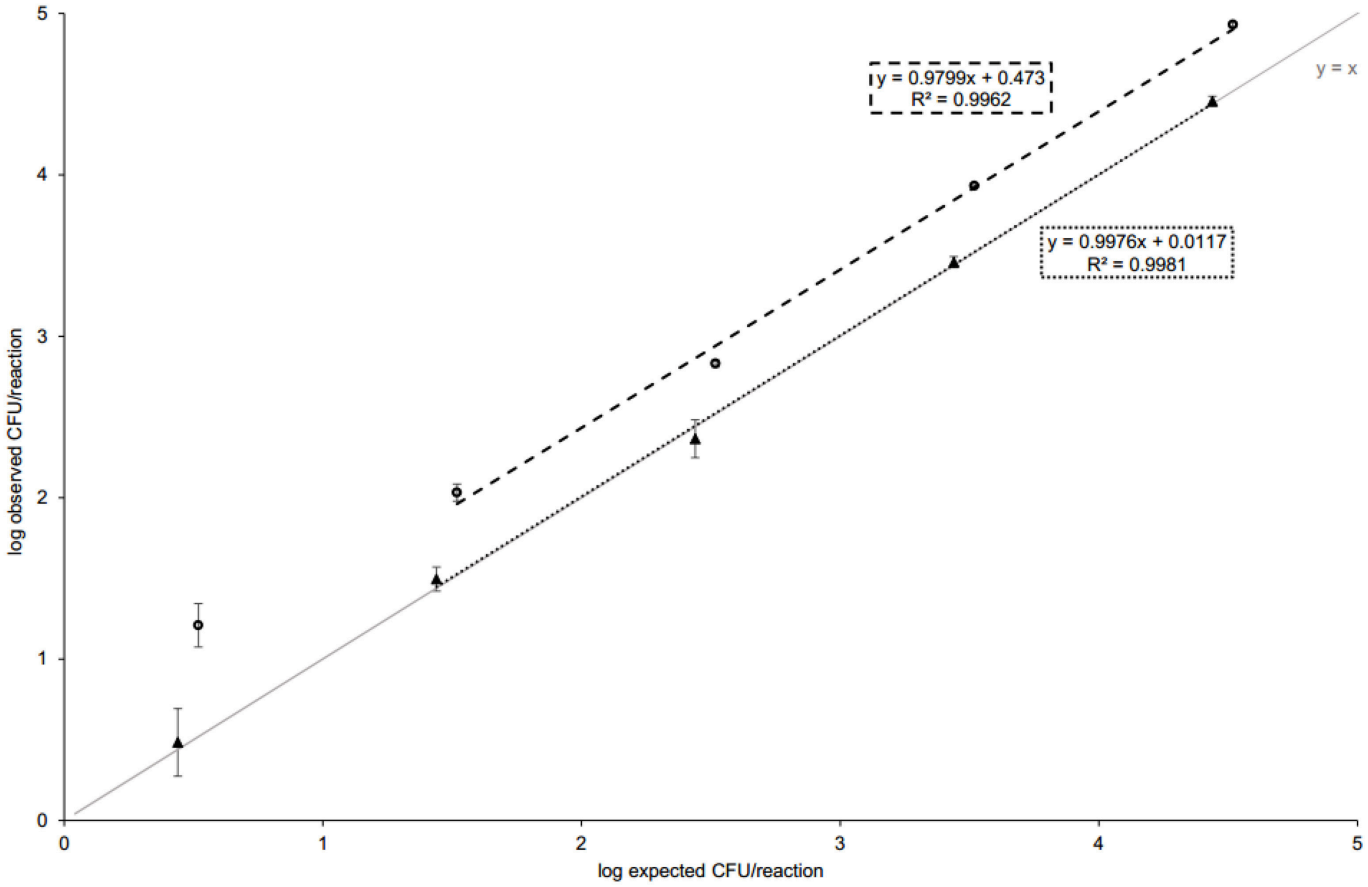
**The performance of qPCR and dPCR**. Standards were prepared and qPCR performed as described in Figure 1. For dPCR, each biological replicate was run on a single chip. The expected values were obtained from plate counting and the solid line represents CFU observed equaled to CFU expected (*y* = *x*). In dPCR, the absolute copy numbers were obtained (absolute quantification), whereas in qPCR, the observed value was calculated from the standard curve (relative quantification). Five 10-fold dilutions from 10^0^ to 10^−4^ are shown. For the calculation of standard curve equation in qPCR, only data belonging to the linear dynamic range was considered (coefficient of variation <33%). In dPCR, same dilutions were used as they all generated precision up to ~10%. For raw data, see Tables 1, 3. Error bars represent standard deviations in qPCR and confidence intervals in dPCR, respectively.

Prior to analyses, Q_T_ and F_T_ values were user-defined at 0.5 and 4,500–6,500, respectively. To evaluate the performance and capability of dPCR for *C. jejuni* quantification, the same standard curve samples were used as for qPCR and values for CFU/ml (or CFU/g after multiplying) were calculated from the obtained results (Table 2). In-house validation of dPCR showed that precision markedly decreased between dilutions 10^−3^ and 10^−4^, showing the same linear dynamic range as in qPCR (Table 1). As the recommended precision for reliable quantification in dPCR is 10%, only dilutions from 10^0^ to 10^−3^ were used for the calculation of trend line (Figure 2). The observed CFU/reaction was plotted against the expected CFU/reaction for both methods—dPCR and qPCR (Figure 2). A trend of overestimation of the observed concentration using dPCR was revealed, whereas the observed qPCR concentrations plotted against the expected values were concordant; the expected values were obtained from the plate count enumeration of *C. jejuni* culture that was used for the preparation of standard DNA dilutions. In dPCR, NTC, and WNTC samples were analyzed for the assessment of analytical specificity (Table 3). Results showed that dPCR generated false positive calls in the negative controls; the average copy number/μl was 4.83 ± 3.65 for WNTC and 3.04 ± 0.48 for NTC. The false positives were in congruence with the overestimation observed over the entire range of tested standard dilutions (Figure 2). Whereas, LOQ and rLOD in qPCR were set to 31 (dilution 10^−3^) and 3 (dilution 10^−4^) observed CFU/reaction, respectively, *C. jejuni* was reliably detected and quantified by dPCR at observed 107 CFU/reaction (dilution 10^−3^, precision 12.98%); at the observed 16 CFU/reaction (dilution 10^−4^), the precision worsened to 36.33%, indicating a wider confidence interval CI (the lower the precision, the tighter the CI). Inferior performance of dPCR could be explained by a high signal-to-noise ratio observed in negative controls.

**Table 2:** **In-house validation of dPCR for *Campylobacter jejuni* absolute quantification**.

**Dilution**	**Expected CFU/reaction**	**Observed CFU/reaction**	**Lower 95% CI**	**Upper 95% CI**	**Precision (%)**
10^−0^	33,000	85,140	82,354	88,022	3.36
10^−1^	3,300	8,544	8,420	8,670	1.48
10^−2^	330	676	645	710	4.89
10^−3^	33	107	95	121	12.98
10^−4^	3.3	16	12	22	36.33
10^−5^	0.33	3	2	7	99.96
10^−6^	0.033	11	7	17	53.37

*Expected CFU/reaction is based on the results of plate count method, assuming no loss during processing. 95% CI values and precision were calculated using QuantStudio 3D AnalysisSuite 3.0.3. SD, standard deviation; CV, coefficient of variation; CI, confidence interval*.

**Table 3:** **Copy number of negative template controls (NTC) and water no template controls (WNTC) per μl of extracted DNA in dPCR**.

**Number**	**NTC (copy number/μl)**	**WNTC (copy number/μl)**
1	5.220	4.831
2	2.008	0.794
3	4.571	0.754
4	4.365	0.908
5	2.769	5.041
6	5.842	5.775
7	0.908	3.428
8	1.488	9.982
9	2.584	11.300
10	2.584	5.526
11	2.758	
12	2.910	
13	1.863	
14	2.703	
Average	3.041	4.834
*SD* average	0.483	3.653
log_10_ average	0.483	0.563

### Comparison of plate count, qPCR, and dPCR

*C. jejuni* was quantified in 75 (60 individual and 15 pooled) broiler neck-skin samples. All samples tested were positive for campylobacters by the plate count method, but 8/75 samples were outside the measuring range as being too numerous to count by the selected sample dilutions (Table 4). All selected *Campylobacter*-suspected colonies were identified as *C. jejuni*. Plate count enumeration showed that *Campylobacter* (*C. jejuni*) contamination ranged from 60 to >15,000 CFU/g of neck skin.

**Table 4:** **Quantification of *Campylobacter jejuni* on 75 naturally contaminated broiler neck-skin samples using the plate count method, qPCR and dPCR**.

**Sample**	**Plate count**	**qPCR**					**dPCR**			
	**[CFU/g]**	**Average Cq**	Pos/all^*^	**Average CFU/g**	***SD* CFU/g**	**CV (%)**	**CFU/g**	**Precision (%)**	**LCL CFU/g**	**UCL CFU/g**
1	940	37.46	3/3	881	267	32.8	3,617	92.2	1,882	6,952
2	1,900	37.90	3/3	733	92	46.6	6,654	58.7	4,192	10,561
3	3,500	35.07	3/3	4,042	1,162	28.8	13,625	38.0	9,872	18,806
4	4,500	34.36	3/3	6,330	1,548	24.5	14,610	35.3	10,797	19,769
5	5,900	33.84	3/3	8,755	1,697	19.4	26,501	26.0	21,035	33,387
6	6,800	34.62	3/3	5,239	396	7.6	25,046	25.6	19,943	31,455
7	2,500	35.32	3/3	3,474	987	28.4	14,213	37.4	10,342	19,534
8	1,600	35.25	3/3	3,565	804	22.6	11,821	41.4	8,359	16,716
9	1,900	37.73	3/3	1,097	17	113.5	8,246	50.5	5,479	12,409
10	3,400	35.23	3/3	3,666	1,175	32.0	18,220	32.3	13,770	24,108
11	90	39.47	2/3	249	119	47.9	381	609.9	53.7	2,708
12	>15,000	**33.11**	**3/3**	**14,092**	**3,439**	**24.4**	60,043	16.7	51,448	70,073
13	9,100	35.19	3/3	3,731	1,107	29.7	10,958	43.0	7,662	15,673
14	12,000	34.18	3/3	6,979	742	10.6	35,873	22.0	29,399	43,772
15	6,900	36.48	3/3	1,608	274	17.0	17,271	33.1	12,976	22,980
16	7,500	38.34	3/3	615	244	67.6	3,743	85.9	2,014	6,958
17	1,400	38.96	2/3	334	90	26.9	3,926	85.9	2,113	7,297
18	3,500	36.78	3/3	1,392	242	14.4	8,184	53.4	5,336	12,552
19	2,900	36.98	3/3	1,158	90	7.8	7,908	53.4	5,156	12,129
20	>15,000	35.64	3/3	2,751	456	16.6	59,412	16.5	51,004	69,206
21	60	39.69	1/3	203	/	/	1,526	166.5	573	4,065
22	11,000	34.76	3/3	4,840	960	19.8	24,251	27.3	19,052	27,286
23	9,000	34.37	3/3	6,371	1,897	29.8	22,885	29.9	17,612	29,738
24	9,000	34.27	3/3	6,736	1,758	26.1	39,155	20.5	32,481	47,202
25	1,900	36.24	3/3	1,862	155	8.3	9,755	45.8	6,690	14,225
26	6,300	39.88	2/3	181	13	7.2	5,425	65.9	3,271	8,999
27	980	37.80	3/3	684	28	4.1	2,904	100.0	1,452	5,807
28	6,600	36.24	3/3	1,925	599	31.1	14,491	35.3	10,709	19,609
29	1,300	36.07	3/3	2,081	203	9.8	6,432	58.7	4,055	10,214
30	12,000	34.89	3/3	4,468	839	18.8	22,255	29.1	17,242	28,724
31	4,800	34.72	3/3	4,940	617	12.5	36,756	21.7	30,214	44,715
32	>15,000	**27.47**	**3/3**	**511,616**	**16,241**	**3.2**	1206,600	3.6	1,164,300	125,400
33	3,800	34.19	3/3	6,985	1,256	18.0	20,524	30.3	15,757	26,733
34	6,600	**33.18**	**3/3**	**13,322**	**2,147**	**16.1**	36,629	22.5	29,892	44,884
35	5,200	33.63	3/3	9,973	1,769	17.7	22,382	29.1	17,341	28,888
36	>15,000	**29.66**	**3/3**	**125,770**	**2,883**	**2.3**	5,425	65.9	3,271	8,999
37	>15,000	**31.13**	**3/3**	**51,022**	**14,532**	**28.5**	2,904	100.0	1,452	5,807
38	3,000	**32.75**	**3/3**	**17,417**	**1,716**	**9.9**	36,529	22.7	29,777	44,812
39	8,100	33.74	3/3	9,351	1,818	19.4	25,970	26.2	20,580	32,771
40	13,000	**32.96**	**3/3**	**15,190**	**401**	**2.6**	40,940	20.4	33,990	49,311
41	2,400	35.99	3/3	2,202	351	15.9	3,866	58.9	2,080	7,186
42	2,100	38.51	1/3	435	/	/	11,465	41.4	8,107	16,212
43	16,000	**32.78**	**3/3**	**17,149**	**2,229**	**13.0**	51,117	18.4	43,182	60,511
44	7,800	34.62	3/3	5,389	1,421	26.4	38,223	21.2	31,540	46,324
45	1,500	37.84	3/3	668	70	10.4	5,317	68.7	3,149	8,978
46	5,000	34.10	3/3	7,401	1,383	18.7	28,091	24.8	22,500	35,071
47	5,300	33.47	3/3	10,968	1,157	10.6	21,304	29.1	16,506	27,497
48	2,600	35.84	3/3	2,408	154	6.4	11,002	43.0	7,692	15,736
49	2,400	36.19	3/3	1,924	199	10.3	7,111	55.0	4,587	11,022
50	5,400	34.33	3/3	6,313	540	8.6	31,439	23.8	25,386	38,935
51	4,800	**32.60**	**3/3**	**19,225**	**2,203**	**11.5**	66,761	31.9	57,418	77,625
52	>15,000	**27.53**	**3/3**	**491,927**	**24,042**	**4.9**	1243,400	3.7	1,199,500	1,288,800
53	3,800	33.62	3/3	9,975	1,419	14.2	19,743	31.2	15,044	25,909
54	6,600	**33.26**	**3/3**	**12,746**	**2,582**	**20.3**	27,523	26.4	21,775	34,789
55	5,200	34.01	3/3	7,781	485	6.2	20,343	30.9	15,542	26,628
56	>15,000	**29.83**	**3/3**	**113,276**	**1,817**	**1.6**	293,570	7.5	273,130	315,530
57	>15,000	**30.95**	**3/3**	**56,985**	**17,574**	**30.8**	175,210	9.7	159,730	192,190
58	3,000	**32.47**	**3/3**	**20,842**	**2,603**	**12.5**	57,757	17.5	49,135	67,890
59	8,100	33.96	3/3	8,016	457	5.7	26,259	27.1	20,667	33,363
60	13,000	**32.42**	**3/3**	**21,689**	**3,571**	**16.5**	70,018	15.8	60,477	81,065
61	5,700	37.17	3/3	262	69	26.4	0	/	0	0
62	650	39.63	3/3	55	17	30.3	715	122.6	321.25	1591.25
63	1,100	38.44	3/3	120	38	37.0	1,026	80.6	568.25	1852.5
64	10,000	37.20	3/3	253	43	16.9	3,493	38.0	2,531	4821.25
65	4,400	37.56	3/3	203	42	20.5	1,098	80.6	608.25	1,983
66	7,800	38.16	3/3	155	18	56.3	22,963	13.7	20,196	26,110
67	3,600	36.96	3/3	300	80	26.6	196	299.9	49	784.5
68	17,000	37.12	2/3	264	20	7.5	97	609.9	13.65	688.25
69	380	37.46	3/3	220	61	33.2	711	109.8	339	1491.75
70	7,400	36.03	3/3	530	28	5.4	1,190	76.1	675.75	2095.25
71	2,000	37.42	3/3	264	66	75.1	872	92.2	453.75	1675.75
72	11,000	36.97	3/3	299	83	27.7	5,608	30.9	4,284	7,340
73	1,000	34.64	3/3	1,292	46	3.5	3,030	42.2	2130.5	4307.75
74	8,600	35.76	3/3	630	20	3.2	1,774	50.0	1144.25	2,749
75	4,200	36.91	3/3	305	46	15.1	1,803	56.8	1,150	2826.5

* *number of positive/all technical replicates. CFU/g, colony forming units/g neck skin; CV, coefficient of variation; LCL, lower confidence level; SD, standard deviation; UCL, upper confidence level. There are included 60 individual (1–60) and 15 pooled (61–75) samples. All qPCR reactions were performed in triplicates (technical replicates) and all samples that showed average CFU below the reliable limit of detection (1,219 CFU/g; 26/75 samples) are shown in gray, whereas all samples that showed average CFU/g above the limit of quantification (12,523 CFU/g; 15/75 samples) are shown in bold. In dPCR, samples were not run in technical replicates and all samples with precision ≥100% (8/75) are shown in gray*.

For qPCR quantification, *C. jejuni* concentration or contamination in the neck-skin samples was calculated from the obtained C_q_ values with the regression-curve equation. All samples were positive by qPCR; however, the majority (60/75) showed values below LOQ (12,523 CFU/g) and 26/75 also below rLOD (1,219 CFU/g). For the latter, the observed cell counts obtained by qPCR ranged from 55 to 1,158 CFU/g (Table 4). In dPCR, 74/75 samples were positive; the negative result was attributed to the sample that was positive by plate count (5,700 CFU/g) and qPCR (262 CFU/g). As the qPCR result showed a value below rLOD, the negative dPCR result could be explained by stochastic variation or sampling error.

For each comparison, samples that were not quantifiable by both methods were discarded from the analysis. Since LOQ selected for only 15/75 quantifiable samples in qPCR and rLOD selected for 49/75, rLOD was taken as a threshold for comparison as all samples above rLOD showed CV <33% (Table 4). In dPCR, precision threshold of 100% was selected to obtain enough data for comparison as precision <10% was rarely observed (Table 4).

**Figure 3:**
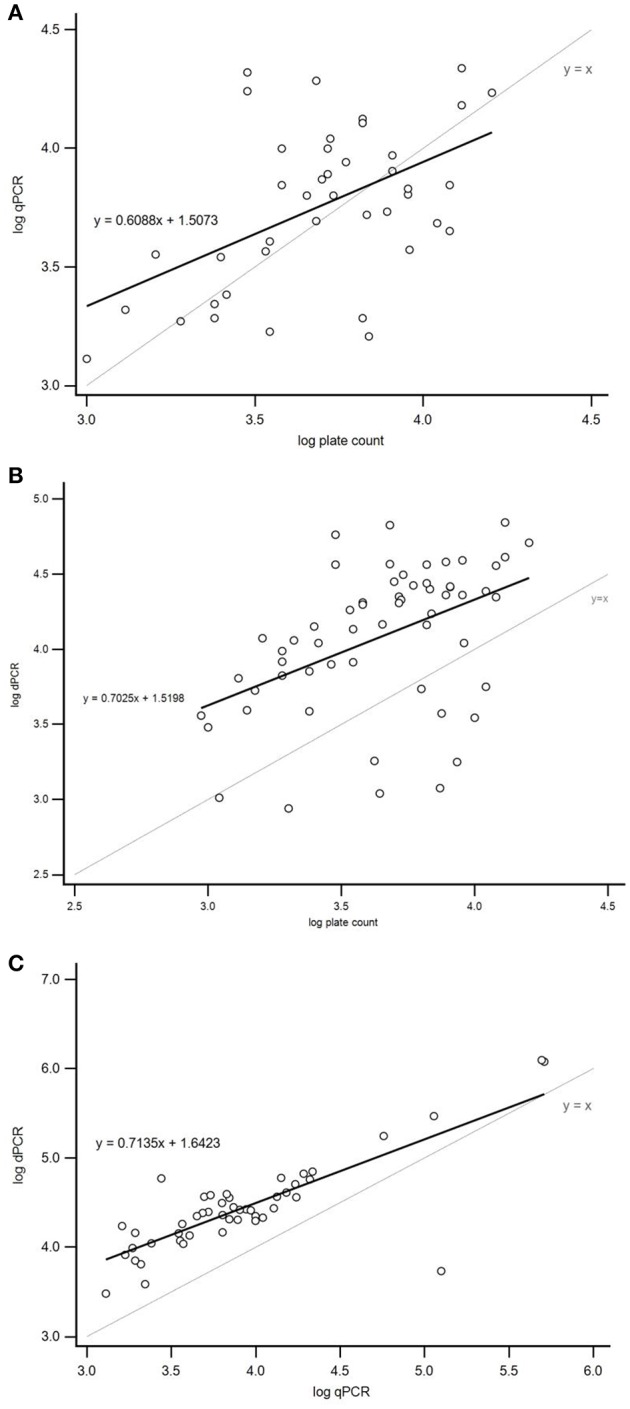
**Linear regression analysis of the log CFU/g numbers observed by the plate count method, qPCR and dPCR for quantification of *Campylobacter jejuni* in 75 naturally contaminated broiler neck-skin samples (15 pooled and 60 individual). (A)** Correlation between qPCR and plate count method: linear trend line equation (thick continuous line). Correlation between variables was moderate and statistically significant (*r* = 0.513; *p* = 0.0006, *N* = 41). **(B)** Correlation between dPCR and plate count method was moderate and statistically significant (*r* = 0.458; *p* = 0.0003, *N* = 59). **(C)** Correlation between dPCR and qPCR was strong and statistically significant (*r* = 0.805, *p* < 0.0001, *N* = 48). Thin continuous lines in all three graphs represent line of equality (*y* = *x*). *r*, Pearson correlation coefficient.

**Figure 4:**
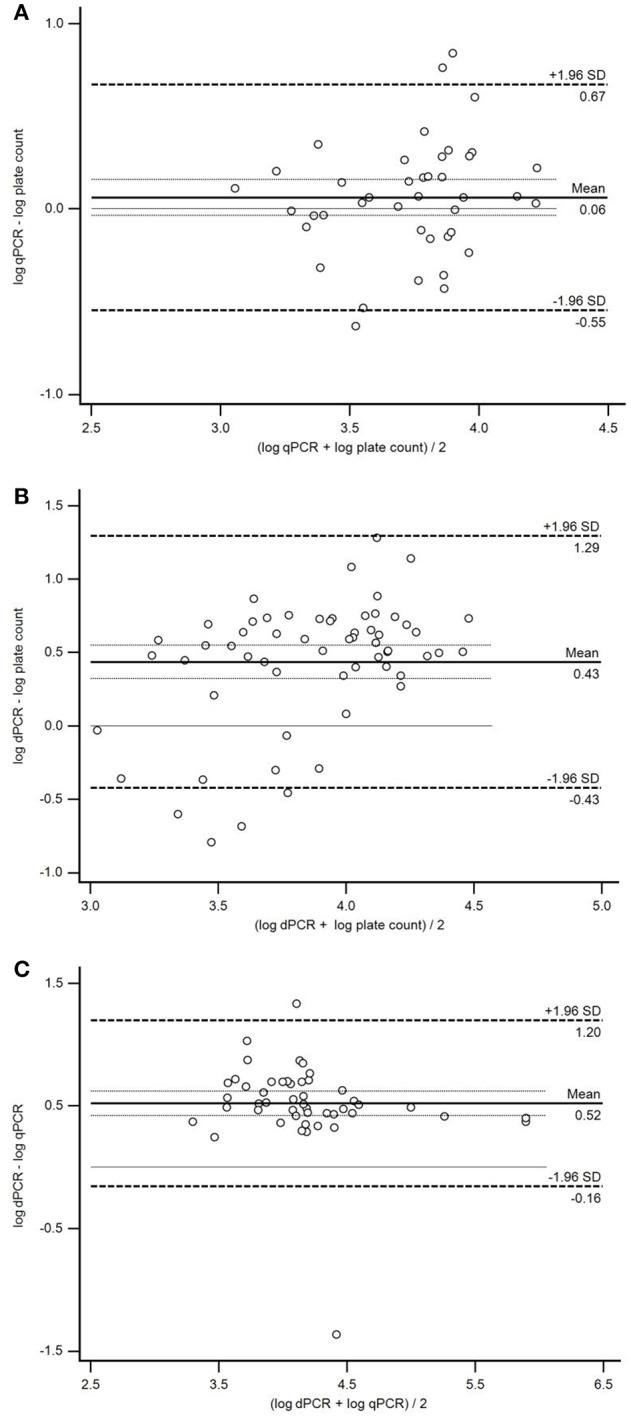
**Evaluation of the agreement between the plate count method, qPCR and dPCR for quantification of *Campylobacter jejuni* in broiler neck-skin samples using Bland–Altman analysis. (A)** Agreement between qPCR and the plate count method. Mean bias 0.06 ± 0.10 log_10_ (95% confidence intervals from −0.55 to 0.67 log_10_, *N* = 41). **(B)** Agreement between dPCR and the plate count method. Mean bias 0.43 ± 0.11 log_10_ (95% confidence intervals from −0.43 to −1.29 log_10_, *N* = 59). **(C)** Agreement between dPCR and qPCR. Mean bias 0.52 ± 0.10 log_10_ (95% confidence intervals from −0.16 to 1.20 log_10_, *N* = 48). Line representing zero log difference is shown as a thin solid line, whereas standard deviations of mean log difference as thin dotted lines. *SD*, standard deviation.

Linear regression analysis demonstrated a moderate and statistically significant linear correlation between qPCR and plate count (*r* = 0.513; *p* = 0.0006) and dPCR and plate count (*r* = 0.458; *p* = 0.0003; Figure 3). Correlation between dPCR and qPCR was strong and statistically significant (*r* = 0.805; *p* < 0.0001; Figure 3). Furthermore, Bland–Altman plots were constructed to assess the agreement between method pairs (Figure 4). In general, the majority of samples were inside the 95% confidence interval limits (±1.96 *SD*); however, 1.96 *SD* values were relatively high in all comparisons ranging from 0.61 (qPCR *vs*. plate count) to 0.86 (dPCR *vs*. plate count). Regarding the mean of differences (log_10_ values), no bias was observed when comparing qPCR and plate count as the average mean of differences was close to the line of equality (difference = 0; Figure 4A). However, when dPCR was involved in the comparison, a significant bias toward overestimation of dPCR was noted in both cases which was probably due to the relatively high number of false positive calls (Figures 4B,C).

## Discussion

*Campylobacter* is one of the most common causes of gastroenteritis worldwide which can also lead to severe post-infection complications such as Guillain-Barré syndrome (Allos, 2001). Fecal contamination during slaughtering, especially at evisceration and plucking phases, is considered to be the major cause of the presence of *Campylobacter* bacteria on broiler carcasses (Corry and Atabay, 2001; Normand et al., 2008). Quick and reliable methods for *Campylobacter* enumeration are of primary interest as they could facilitate food surveillance and enable the assessment of control measures taken to reduce *Campylobacter* contamination in the food-producing environment.

Currently, the ISO 10272-2:2006 plate counting method is the only accepted and standardized quantification method for campylobacters in various matrices. However, molecular-based approaches could provide laboratories with more rapid procedures for quantification, if proven satisfactory regarding the reproducibility and repeatability for the selected types of samples, in addition to adequate limits of quantification and detection. However, discrimination of viable and damaged or dead *Campylobacter* cells with molecular methods remains challenging.

Results of the present study suggest that PCR-based methods require a relatively high *Campylobacter* contamination of the neck skin for reliable detection and quantification when using the sample preparation described. The employed qPCR assay performed to the best of theoretical limits with rLOD of three target copies per reaction (Bustin et al., 2009); however, the preceding sample dilutions markedly impaired *C. jejuni* detection and quantification at the proposed contamination levels 500–1,000 CFU/g (EFSA, 2011). This renders qPCR and dPCR unable to reliably quantify *C. jejuni* in the range of the proposed microbiological criteria for *Campylobacter* in foodstuffs, but with a modification of the protocol for sample preparation (e.g., elimination of the initial 10-fold dilution during sample homogenization), a more concentrated input material could be obtained for DNA extraction. In addition, by using smaller volumes for elution of DNA or inhibitor-resistant DNA polymerase, qPCR could further be optimized for reliable detection at the proposed contamination levels. Namely, higher concentrations of inhibitors and lower DNA yield due to the incomplete recovery of the cells may occur in environmental samples and PCR inhibitors were also observed in broiler neck skin (Josefsen et al., 2010; Schnider et al., 2010). However, as in the present study inhibition was not observed in qPCR standards prepared with spiking the negative matrix, it is not expected in the naturally contaminated samples. Moreover, the employed DNA extraction kit is regularly used at our laboratory for bacterial DNA extraction from complex samples and was extensively tested and optimized to enable maximal removal of inhibitors. In addition, its performance was compared to one of the commercially favored extraction kits which are specially designed for demanding extractions and purifications of bacterial DNA from food samples, including special matrices with a high proportion of PCR inhibitors. Comparison showed that the kit which was employed in the present study led to significantly (*p* = 0.0153) higher *C. jejuni* counts observed in qPCR (Papić et al., 2016). The detection rate could be increased by adding the enrichment step; however, this impedes the enumeration of the pathogen. Although the inhibitors present in the sample are diluted in the enrichment step, the enrichment media itself may include PCR inhibitors (Josefsen et al., 2004).

Previously, *Campylobacter* spp. has been quantified in the poultry slaughterhouse water samples by the plate count method, droplet dPCR (ddPCR), and qPCR; both molecular-based methods outperformed the culture-based method commonly used in the testing of poultry processing water samples (Rothrock et al., 2013). Cremonesi et al. (2016) quantified the common foodborne pathogens including *Campylobacter* spp. in the soft cheese using ddPCR and qPCR, discovering higher sensitivity of ddPCR. In our study, inferior performance of dPCR could be explained by the high signal-to-noise ratio detected in negative controls. Similar results have also been observed previously (Bosman et al., 2015; Luedtke and Bosilevac, 2015). Other authors, however, reported that dPCR surpasses qPCR both in terms of precision and sensitivity (Strain et al., 2013; Taylor et al., 2015). Another advantage of dPCR is that it is generally less prone to PCR inhibition (Rački et al., 2014). In the present study, the quantification of low target samples was challenging due to false positive wells. Discrimination between false positive and true positive samples with dPCR proved difficult as WNTC and NTC negative controls all regularly showed up to 11 target copies/μl despite using a stringent and run-adapted fluorescence threshold. Therefore, samples may be classified as false positive; such samples might also explain the overestimation of observed concentration by dPCR in comparison to the plate count method and qPCR.

When the observed log_10_ CFU/reaction were plotted against the expected log_10_ CFU/reaction, an overestimation of observed cell counts was noted in dPCR. As the average log_10_ value for both NTC and WTNC was similar (0.48 and 0.56, respectively) to the *y*-intercept value of dPCR trend line (0.47) in Figure 2, this could explain the overestimation of dPCR observed *vs*. expected log_10_ CFU/reaction. The overestimation was observed over the entire 10-fold dilution range, which is not surprising as the obtained copy number/μl is multiplied by total DNA elution volume and sample dilution to get the final result, multiplying the false positive effect. The same phenomenon of dPCR overestimation was observed in the naturally contaminated samples as both Bland–Altman comparisons involving dPCR showed bias (Figures 4B,C). On the other hand, qPCR was generally concordant with the plate count method for the *C. jejuni* culture dilutions as the observed cell counts correlated with the expected cell counts. False positive samples can be observed both in chip-based dPCR and ddPCR, and might not always be identified based on the fluorescence data as they are often well-separated from the true negative events (Strain et al., 2013; Bosman et al., 2015). Signal-to-noise ratio could be further assessed analyzing a larger number of negative control samples. Also, low-target samples could be more reliably analyzed by running more parallels, increasing the DNA template volume or optimizing the dPCR assay (e.g., designing new primers and/or probes). However, due to the time requirement and relatively high reagent cost of chip-based dPCR, this would not be suitable for routine detection and quantification of *C. jejuni* in the food industry. When comparing costs of the three methods employed, the plate count method was found to be approx. twice as expensive as PCR-based methods, whereas dPCR and qPCR were comparable when regarding cost per sample. However, qPCR enables marked decrease of costs with higher throughput and quicker turnaround time, in addition to the possibility of decreasing the reaction volume.

The observed inter-method differences in the present study could be explained by different methodology and limitations of each method. All three quantification methods were generally interchangeable as shown by Blant–Altman analysis; however, they displayed a relatively high mean bias. This increase in variation was probably due to stochastic effects and sampling errors which occur in samples with low target concentration (i.e., naturally contaminated neck skin; Irwin et al., 2010). As mentioned above, in qPCR, we alleviated this effect by processing more replicates of one sample and excluding from the analysis all samples that were negative in at least one replicate or below rLOD. Such approach of analyzing more replicates could also be expanded to dPCR and plate count method; however, this would render the methods unsuitable for routine use due to significant time and financial investment.

Albeit time consuming, the plate count method is the gold standard for enumeration of *Campylobacter* spp. on broiler skin. However, not all cells can be recovered by conventional cultivation techniques due to special growth requirements and VBNC state. Another important limitation of the plate count method is the inability to distinguish between different species without additional identification steps. Even though *C. jejuni* is globally responsible for more than 85% of human infections and is the most frequently isolated species in poultry samples, other (non-*jejuni*) pathogenic campylobacters such as *C. coli* and *C. lari* should not be neglected (Shane, 2000; Jørgensen et al., 2002; Mason et al., 2013). Prevalence of *C. coli* was found to be as high as 40% of *Campylobacter*-positive neck-skin samples when quantifying *C. jejuni* and *C. coli* in broiler neck-skin samples using qPCR (Schnider et al., 2010) and co-infection with different *C. jejuni* strains or even with different *Campylobacter* species was also observed in human patients (Linton et al., 1997; Godschalk et al., 2006). Although, in our case, all isolates were identified as *C. jejuni*, a possibility of a mixed contamination cannot be ruled out. Generally, PCR-based quantification methods are more specific than plate counting.

Molecular methods for pathogen detection and quantification are quick, selective and precise; however, the inability to differentiate between viable and dead cells hampers their use in food industry. Another important limitation of pathogen quantification in naturally contaminated samples, such as broiler neck skin, is low concentration of target organisms which can impede reliable detection and quantification. When comparing two or more methods, this stochastic effect, leading to increased variation between replicates, impedes the assessment of inter-method agreement (Irwin et al., 2010). Furthermore, reproducible quantification of low abundance targets (<1,000 target copies/PCR) in complex samples by qPCR is difficult due to the inherent differences in the amplification efficiency between individual templates in the amplifying DNA population, known as the Monte Carlo effect (Karrer et al., 1995). This may also contribute to the occurrence of wells and droplets with intermediate fluorescence in dPCR and ddPCR, respectively (Dreo et al., 2014).

CFUs can be deceptive for quantification of the infection risk especially in the case of fastidious microorganisms such as campylobacters, since the stressed and VBNC cells probably also pose a health risk to consumers (Josefsen et al., 2010). New PCR-based strategies, collectively known as molecular viability analyses, promise to overcome this obstacle as they are able to differentiate nucleic acids associated with viable cells from those associated with inactivated cells (reviewed in Cangelosi and Meschke, 2014). Treatment of samples with ethidium monoazide (EMA) in combination with (q)PCR reportedly enables differentiation between live and dead campylobacters (Rudi et al., 2004, 2005). Josefsen et al. (2010) developed a qPCR assay in combination with prior propidium monoazide (PMA) sample treatment to differentiate between live and dead cells on chicken skin. However, efficiency of such viability PCR techniques depends on a complex set of parameters including experimental, target and sample features (Fittipaldi et al., 2012). This was confirmed by Pacholewicz et al. (2013), who reported that PMA treatment of the samples prior to qPCR did not fully reduce the signal from dead cells, possibly due to a high bacterial load in samples. Krüger et al. (2014) proposed the use of reliable quantification of intact and potentially infectious units (IPIU) of *Campylobacter* spp. for the assessment of infection risk using qPCR. For this purpose, the authors suggest implementation of a robust viability PCR that includes carefully chosen samples process control for each quantification setup (Krüger et al., 2014).

In conclusion, qPCR outperformed dPCR in quantification of *C. jejuni* in the poultry neck-skin samples and exhibited the LOQ of 31 CFU/reaction (12,523 CFU/g). However, none of the molecular-based methods enabled its reliable quantification at the proposed microbiological criteria of 500–1,000 CFU/g. This, at least according to results of the present study, renders the available molecular-based methods unsuitable for quantification of such samples and calls for the improvement of sample preparation steps and/or development of improved or novel analytical methods for direct *Campylobacter* enumeration in poultry samples. Optimization of molecular-based methods to the level at which they would enable reliable *Campylobacter* quantification in poultry samples with low contamination would substantially reduce both the time and cost requirements as well as facilitate *Campylobacter* surveillance throughout all stages in the food production chain.

## Author contributions

Design of the study: BP, UH, MP, IG, MB, MO, and DK; Sample collection: IG, UZ, and MO; Classical bacteriology: IG, UH, and MB; Molecular methods: BP, MP, UH, UZ, and DK; Data analysis: BP and DK; Manuscript preparation: BP, MP, IG, and DK. All authors read and approved the final manuscript.

## Funding

This study was funded by the Slovenian Research Agency and the Ministry of Agriculture and Environment of the Republic of Slovenia (Grants V4-1110 and J4-7608).

### Conflict of interest statement

The authors declare that the research was conducted in the absence of any commercial or financial relationships that could be construed as a potential conflict of interest.
